# Proofreading mechanisms of the innate immune receptor RIG-I: distinguishing self and viral RNA

**DOI:** 10.1042/BST20230724

**Published:** 2024-06-17

**Authors:** Mihai Solotchi, Smita S. Patel

**Affiliations:** 1 Department of Biochemistry and Molecular Biology, Robert Wood Johnson Medical School, Rutgers University, Piscataway, NJ 08854, U.S.A.; 2 Graduate School of Biomedical Sciences, Robert Wood Johnson Medical School, Rutgers University, Piscataway, NJ, U.S.A.

**Keywords:** helicase, nucleic acid receptors, RIG-I like receptors

## Abstract

The RIG-I-like receptors (RLRs), comprising retinoic acid-inducible gene I (RIG-I), melanoma differentiation-associated gene 5 (MDA5), and laboratory of genetics and physiology 2 (LGP2), are pattern recognition receptors belonging to the DExD/H-box RNA helicase family of proteins. RLRs detect viral RNAs in the cytoplasm and respond by initiating a robust antiviral response that up-regulates interferon and cytokine production. RIG-I and MDA5 complement each other by recognizing different RNA features, and LGP2 regulates their activation. RIG-I's multilayered RNA recognition and proofreading mechanisms ensure accurate viral RNA detection while averting harmful responses to host RNAs. RIG-I's C-terminal domain targets 5′-triphosphate double-stranded RNA (dsRNA) blunt ends, while an intrinsic gating mechanism prevents the helicase domains from non-specifically engaging with host RNAs. The ATPase and RNA translocation activity of RIG-I adds another layer of selectivity by minimizing the lifetime of RIG-I on non-specific RNAs, preventing off-target activation. The versatility of RIG-I's ATPase function also amplifies downstream signaling by enhancing the signaling domain (CARDs) exposure on 5′-triphosphate dsRNA and promoting oligomerization. In this review, we offer an in-depth understanding of the mechanisms RIG-I uses to facilitate viral RNA sensing and regulate downstream activation of the immune system.

## Introduction

RIG-I-like receptors (RLRs) are crucial components of the innate immune system of vertebrates. The RLR family encompasses three proteins: RIG-I (retinoic acid-inducible gene I, DDX58), MDA5 (melanoma differentiation-associated gene 5, IFIH1), and LGP2 (laboratory of genetics and physiology 2, DHX58). These nucleic acid receptors are present in the cytoplasm and serve as first responders to viral infections [1–5]. When RNA viruses invade host cells for replication, they expose their viral RNA genomes and generate double-stranded RNA (dsRNA) replication intermediates, which the RLRs recognize as foreign molecules [6,7]. Upon recognition of viral RNA, RIG-I, and MDA5 interact with the mitochondrial antiviral signaling protein (MAVS) and promote its oligomerization on the mitochondrial membrane [8–10]. These MAVS oligomers facilitate the phosphorylation of IRF3 and IRF7, inducing the expression of type I interferons (IFN-α and IFN-β), cytokines, and chemokines. The activated MAVS oligomers also recruit IKKα, IKKβ, and IKKγ in the NFκB pathway to induce the expression of various pro-inflammatory genes, such as TNF-α, IL-6, and IL-1β, and chemokines [11,12]. The IFN response establishes an antiviral state by up-regulating IFN-stimulated genes (ISGs), which collectively limit viral replication by targeting various steps in the viral life cycle [13,14].

The innate immune response to viral infection is essential for activating the adaptive immune response, which helps defend against future infections [15]. The IFNs up-regulate the expression of MHC class I molecules on infected and neighboring cells, thereby enhancing antigen presentation of dendritic cells (DCs) to CD4^+^ and CD8^+^ T cells [16]. Type I IFN activities drive both T cell and B cell activation, which aid in the cytotoxic activities of natural killer (NK) cells and the production of virus-specific antibodies. The pro-inflammatory cytokines play a critical role in orchestrating the early immune response by recruiting and activating additional immune cells, including DCs, to the site of infection [15]. This recruitment is essential for effective antigen presentation. Furthermore, the cytokines help to differentiate naïve T cells into Th1 cells, and to enhance the maturation of antigen-presenting cells, thereby facilitating the stimulation of the adaptive immune system.

While the up-regulation of IFNs promotes the resolution of viral infections, prolonged or excessive activation of the immune response after viral infection or aberrant activation of IFN signaling by endogenous RNAs can lead to sustained production of pro-inflammatory cytokines, which can be a risk factor for inflammatory diseases such as atherosclerosis, rheumatoid arthritis, multiple sclerosis, asthma, and COPD [12,17–19]. Therefore, the antiviral response must be tightly regulated and the RLRs must faithfully distinguish viral RNAs from the vast pool of endogenous RNAs [20–22]. Host cells utilize mechanisms involving RNA modifications to ensure that RLRs do not bind to endogenous RNAs; often, viruses hijack such evasion mechanisms to avoid detection by the RLRs [23]. Striking the balance between effective viral RNA recognition and endogenous RNA avoidance is crucial for orchestrating an effective antiviral response while preventing harmful reactions to the host. The RLRs have an intricate multistep mechanism for RNA recognition and proofreading, best understood in RIG-I. This review aims to complement the existing knowledge surrounding RIG-I's cellular characteristics and phenotypes reviewed in the literature [3,4,10,24–28] with a deeper dive into the biochemical and structural mechanisms of RIG-I activation and RNA proofreading.

## Modular structure of RLRs

**Figure 1.**
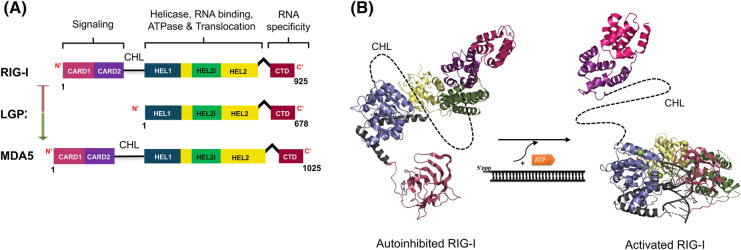
Structural architecture of the RIG-I-like receptors (RLRs). (**A**) Comparison of domain architecture of the RIG-I-like receptor family. The green arrow indicates activation of MDA5, and the red indicates inhibition of RIG-I. (**B**) Activation model of RIG-I. 5′ppp dsRNA and ATP binding induce a conformational change from autoinhibited (PDB: 4A2W) (CTD modeled from PDB: 2YKG) to the activated state (PDB: 5F9H). The exposure of N-terminal CARDs marks RIG-I activation.

The RLR proteins belong to the DExD/H-box family of RNA helicases and share a multidomain architecture [29–32] (Figure 1A). The recA-like helicase domains (Hel-1, Hel-2) are centrally located with a nested insertion domain (Hel2i) and a flanking RNA recognition domain at the C-terminal end (CTD). The N-terminal end of RIG-I and MDA5 contain tandem caspase activation and recruitment domains (CARDs), essential for initiating immune responses upon RNA binding [2]. LGP2 lacks these CARDs; hence, it cannot trigger an immune response directly upon RNA binding, but LGP2 plays a key role in regulating the activities of RIG-I and MDA5. The flexibly linked domains allow the RLRs to switch between different conformational states in response to RNA binding [32,33].

The CTD and the helicase domains provide the RNA-binding functions, while ATPase activity at the Hel-1 and Hel-2 interface controls the outcomes of the RNA interactions [34]. The ATPase site is only activated when the RLRs are bound to RNA and it promotes translocation of the RLRs on RNA [35–38]. In MDA5, this activity controls filament assembly and disassembly on long dsRNAs [36]. In RIG-I, this activity helps proofread for erroneous RNA binding, contributing to selective responses to viral RNAs [37,39–42] as discussed below. The CARD domains of RIG-I and MDA5 are tethered to Hel-1 by 50–100 amino acids long intrinsically disordered linkers, CARDs-helicase linker (CHL), that serve crucial regulatory functions in RIG-I, as discussed below.

Despite their structural similarities, the RLRs have distinct roles in recognizing and responding to viral RNAs. RIG-I and MDA5 complement each other by recognizing different RNA features in viral RNAs — 5′-triphosphate (5′ppp) RNA ends versus long dsRNA regions — enabling the detection of diverse virus classes [43–45]. LGP2, which lacks CARDs, appears to be the dark horse of the family; it is essential in activating MDA5 and providing a negative feedback mechanism to silence both RIG-I and MDA5 [2,5,38,46,47].

## Sequestering CARDs: RIG-I's autoinhibition mechanism

The CARDs are the signaling domains of the RLRs that interact with the MAVS to activate the RIG-I signaling pathway [8,9,48]. Isolated CARDs of RIG-I and MDA5 can stimulate the RIG-I signaling pathway without the presence of viral RNA [2,49–52]. This emphasizes the necessity of establishing mechanisms to keep CARDs autoinhibited in the absence of a viral infection. The CARDs autoinhibition mechanism is well characterized in RIG-I but remains unknown in MDA5. The crystal structure of duck RIG-I showed that in the absence of RNA, the Hel2i domain sequesters the CARDs through extensive interdomain interactions, rendering them inaccessible for interactions with downstream adapter proteins [32] (Figure 1B). In this autoinhibited conformation, the helicase domains adopt an open domain conformation that can bind ATP, but the ATPase active site at the interface of Hel-1 and Hel-2 is not catalytically competent for hydrolysis [34]. The intrinsically disordered linker between CARDs and Hel-1 (CHL), not resolved in the structure, plays a crucial role in stabilizing the autoinhibited state, and small deletions in CHL lead to CARDs exposure and constitutive RIG-I signaling [53].

When RIG-I binds to dsRNA, the helicase domains shift into a closed conformation. This involves a significant rotation of the Hel2i and Hel2 domains, which encapsulates the dsRNA and brings Hel2i closer to the CTD [30–32,34] (Figure 1B). The rotational conformational change breaks the CARD2:Hel2i interactions, exposing the CARDs and CHL to the solution (Figure 1B). CARDs exposure has been studied by hydrogen-deuterium exchange and mass spectrometry (HDX-MS) [53–56]. The solvent accessibility as measured by deuterium exchange in the CARDs increases upon binding to 5′ppp dsRNA and further increases upon the addition of the non-hydrolyzable ATP analog, ADP.AlF_3_, that mimics the transition state during ATP hydrolysis [54]. This pattern of CARDs exposure by RNA binding and enhancement by ADP.AlF_3_ was observed by FRET [57].

## Distinct RNA binding specificities of RIG-I, MDA5, and LGP2

**Figure 2.**
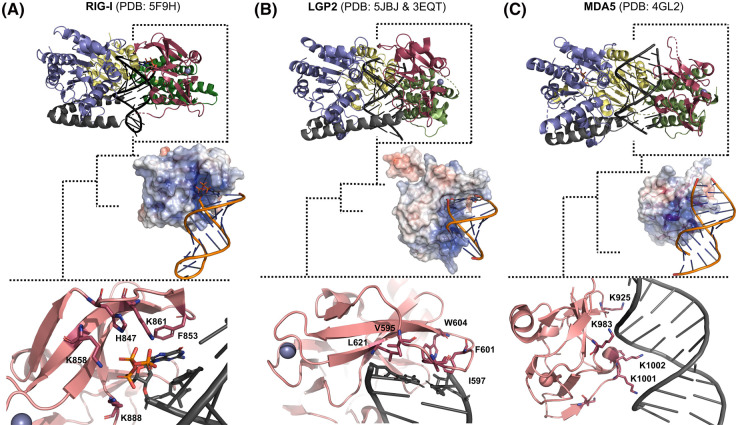
The CTD of RLRs determines their RNA binding specificities. The RNA-bound states of RIG-I, MDA5, and LGP2 show similar structure and RNA footprint within the helicase domains’ channel. The specificity of the RLRs arises from their C-terminal domain (brick red), distinguished by a conserved positively charged patch, as depicted by the blue regions in the electrostatic surface. (**A**) RIG-I (PDB: 5F9H) uses positively charged residues in the capping loop to form strong interactions with the 5′-triphosphate of dsRNA, while base-stacking interactions with aromatic F residues form a preference for blunt-ended RNA. (**B**) LGP2 (chicken full-length PDB: 5JBJ) and (human CTD PDB: 3EQT) has a hydrophobic capping loop that facilitates blunt end RNA binding. (**C**) MDA5 (PDB: 4GL2) does not have RNA end-binding preference. Residues from the positively charged patch on MDA5's CTD interact with the backbone of the RNA and within the minor groove.

RLRs have similar footprints on RNA, spanning 10–14 base pairs (Figure 2), but they exhibit different RNA length and RNA end requirements for high-affinity binding. RIG-I and LGP2 bind to short dsRNAs with blunt ends as monomers with high affinity; in contrast, a monomer of MDA5 has a weak affinity for dsRNA and needs to multimerize to bind strongly to dsRNA [31,58–60]. Multiple RIG-I molecules can bind to longer dsRNA with a 5′ppp and blunt end facilitated by the end binding and ATPase-driven translocation mechanism [37,61]. When multiple RIG-I are bound, the CTD caps the 5′ppp end in the end-bound molecule and adopts a distinct conformation to engage with the RNA backbone in the internally bound molecules, similar to the conformation observed in MDA5 [62]. MDA5 binds to long dsRNA cooperatively, forming filaments. Cryo-EM studies show that the multimers are stabilized by specific subunit interactions involving hydrophobic residues of Hel-1 and the C-terminal tail of the CTD of one subunit interacting with pincer helices in the adjacent subunit [60]. Such interactions are not observed in RIG-I oligomers on dsRNA [62,63]. LGP2 does not form higher oligomers on dsRNA [38] as observed with RIG-I.

The CTD, comprising ∼125 amino acids plays a pivotal role in determining the RNA-binding preferences of the RLRs [64–66]. In contrast, the helicase domains bind RNA non-specifically, interacting with the ribose and phosphate backbone [30–32]. The CTD shares a common global fold among the RLRs and features a positively charged RNA-binding patch (Figure 2). The preference for RNA binding is determined by a capping loop in the CTD. This loop contains specific residues that play a role in the distinct RNA specificities of the RLRs. RIG-I's CTD has a high affinity for dsRNA blunt ends bearing a 5′ppp or 5′-diphosphate (5′pp) moiety [7,43]. This end-binding preference in RIG-I is conferred by a conserved set of basic residues (K858, K861, K888, and H847) in the capping loop, which coordinate with the 5′ppp/pp (Figure 2A). The blunt end specificity of RIG-I is due to the aromatic residues F853 and F856 in the CTD that stack with the blunt end. Mutations of these residues abolish 5′ppp dsRNA-induced immune stimulation [66]. In contrast, MDA5 does not exhibit a specific preference for RNA ends; its capping loop interacts with the stem region of the RNA in the minor groove [48,58] (Figure 2C). LGP2, like RIG-I, prefers the blunt end but lacks affinity for a 5′ppp [38,58]. LGP2's capping loop contains hydrophobic amino acids (V595, I597, F601, W604, L621) that facilitate binding to blunt end RNA (Figure 2B).

Both short 10–14 bp hairpin RNAs and long dsRNA with 5′ppp and blunt end activate RIG-I signaling [67]; intriguingly, this capability does not extend to ∼10 bp duplex RNAs [68,69]. In the context of non-hairpin duplex RNAs, RIG-I requires at least 20 bp of dsRNA to activate the signaling pathway [66]. On the other hand, MDA5-mediated IFN signaling requires dsRNAs > 0.5 kbp in length [45].

## Dual role of the intrinsically disordered CARDs-helicase linker of RIG-I

**Figure 3.**
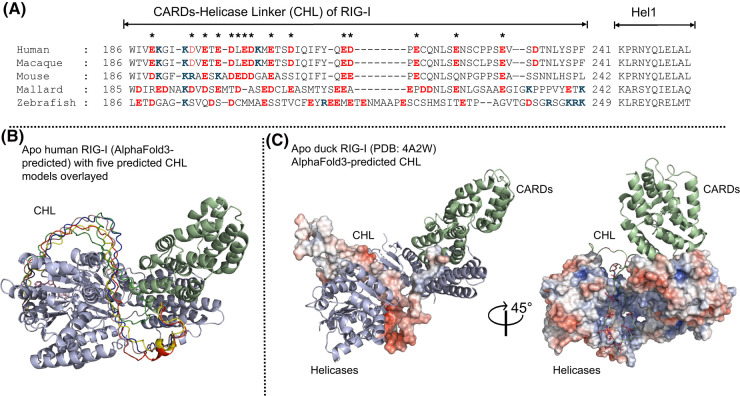
RIG-I's intrinsically disordered, charge-conserved CHL provides an RNA-gating mechanism. (**A**) RIG-I sequences spanning residues ∼180–280 are aligned from various vertebrates. While sequence conservation in the CHL is not as pronounced as in the Helicase domains, the prevalence and conservation of negatively charged residues suggest their significant functional role in RIG-I regulation. Glutamate and aspartate residues are colored red, and lysines are blue. Asterisks (*) indicate positions where a charge is conserved in the sequence across species. (**B**) The AlphaFold3 predictions of full-length human apo RIG-I render the CHL within the Helicase RNA-binding channel. The top 5 ranked predictions of the CHL are overlayed on the top rank prediction of the full-length protein to emphasize the confidence of spatial fitting for the CHL, rather than the weak confidence score of the region's folding propensity. The CTD of RIG-I has been hidden to reveal the CHL predictions, but it is predicted to sit between Hel1 and the CARDs at the end of the V-linker. (**C**) The electrostatic surface of the autoinhibited duck RIG-I Helicase domains (PDB: 4A2W) displays a positively charged RNA-binding channel. AlphaFold and biochemical studies predict that the negatively charged CHL will reside in this channel when RNA is absent. The electrostatic surface of the predicted CHL displays a high degree of charge complementation to the Helicase electrostatics.

**Figure 4.**
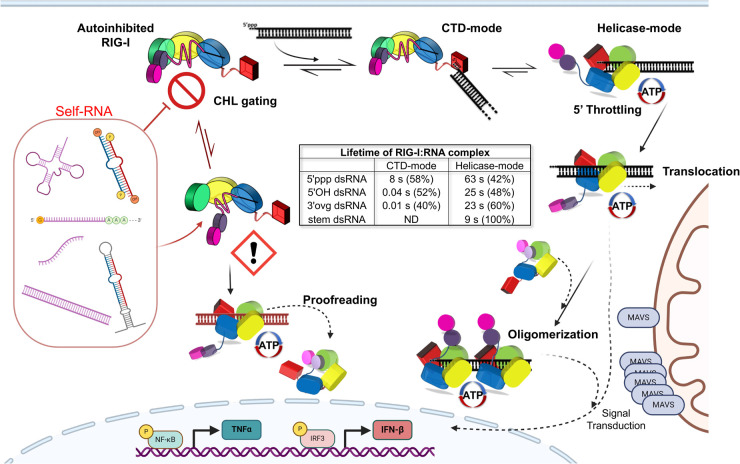
RIG-I's activation pathway with ordered RNA binding and ATPase proofreading. Top left, RIG-I initiates its cycle in the autoinhibited state, characterized by CARD-Hel2i sequestration and CHL (pink) gating the Helicase's RNA binding site to prevent nonspecific RNA binding. The CTD (red) is free to interact with RNA and forms high-affinity interactions with 5′ppp dsRNA ends, a characteristic feature of viral RNA replication intermediates, resulting in the CTD-mode conformation. The left side of the figure shows binding to non-specific RNA and the right side shows binding to viral RNA. The viral RNA with a 5′ppp RNA end has a longer lifetime on the CTD compared with non-ppp RNAs, as indicated in the center table that tabulates the kinetic lifetime measurements of RIG-I CTD- and Helicase-mode. This prolonged lifetime facilitates the activated Helicase-mode conformation, where the RNA is bound to the Helicase, and CARDs are free. The ATPase activity induces translocation-mediated oligomerization, clustering multiple RIG-I molecules on one RNA, thereby enhancing the likelihood of CARD oligomerization. Strong CTD interactions with 5′ppp prolong the activated state's duration by inhibiting RIG-I's translocation. The CARDs oligomers transmit their signal by interacting with downstream partner MAVS, establishing an antiviral and pro-inflammatory state through the up-regulation of TNF-α and IFN-β. Ultimately, RIG-I's ATPase activity dissociates and inactivates the complex. If RIG-I binds to a nonspecific RNA end, weak association with CTD results in rapid dissociation. If RIG-I's gating mechanism is compromised, self-RNA regions can bind to the Helicase directly. In the absence of a 5′ppp-mediated throttling effect, however, RIG-I's ATPase and translocation processes rapidly dissociate such self-RNAs, minimizing aberrant RIG-I activation.

The CARDs in RIG-I and MDA5 are connected to Hel-1 by an intrinsically disordered region. This CHL is highly acidic, spanning ∼56 amino acids in RIG-I and ∼100 amino acids in MDA5; LGP2 does not contain this region. Despite the lack of evolutionary conservation in the CHL sequence among homologs relative to the helicase domain, the negative charges within the CHL of both RLRs are well conserved (Figure 3A). Recent studies have revealed a crucial regulatory role of RIG-I's CHL region in stabilizing the CARD2:Hel2i interface, thereby keeping the CARDs autoinhibited [53]. Accordingly, small deletions or mutations resulting in charge-flipping lead to partial exposure of the CARDs and immune signaling without viral RNA. While the CHL remains unresolved in structures, AlphaFold3 predictions fit the CHL within the RNA-binding channel of the Helicase domains, and surface electrostatics highlight the charge complementarity [70,71] (Figure 3B,C). Biochemical studies provide evidence that the acidic CHL plays an RNA-gating role by blocking the RNA-binding pocket of the helicase domain to deter non-specific RNA binding [53] (Figure 4). Deletion of the CHL and CARDs-CHL disables this RNA-gating mechanism, and the truncated RIG-I constructs bind avidly to non-triphosphate and non-blunt-ended RNAs as well as single-stranded RNAs with nanomolar *K*_D_ values [53]. Any such gating role of the CHL in MDA5 has not yet been established.

## RNA-binding mechanism of RIG-I

With the CHL gating mechanism blocking RNA access to the helicase domains, RNA can only bind to RIG-I at the CTD domain. Thus, the CHL gating mechanism in RIG-I ensures an ordered and regulated RNA-binding mechanism, wherein the helicase domains only engage with RNAs that the CTD binds to (Figure 4). The CTD of RIG-I is flexibly linked to the helicase domains and can bind RNA independently and specifically [65,66]. Biochemical studies show that the isolated CTD of RIG-I binds 5′ppp blunt-ended RNAs with an affinity similar to the full-length RIG-I [37,68,72]. The high specificity of the CTD for 5′ppp blunt-ended RNA ends, and its flexibility relative to the helicase domains provide a mechanism for RIG-I to efficiently survey the cytoplasm for viral RNAs. Stopped-flow kinetic studies show that CTD specificity is due to its different off-rates from specific and non-specific RNAs. The CTD binds to 5′ppp or non-specific RNA ends indiscriminately with a fast on-rate, but its off-rate depends on the type of RNA end [37]. Non-ppp RNAs display fast on and off-rates resulting in their short lifetimes on RIG-I CTD (Figure 4). In contrast, the 5′ppp blunt end RNA shows a fast on-rate but a slow off-rate, forming a long-lived CTD complex (Figure 4). The long-lived CTD-bound RNAs is loaded into the helicase domain more efficiently while the short-lived RNAs dissociate from the CTD. Thus, RIG-I has two conformational states on 5′ppp dsRNA: a CTD-mode conformation, where the dsRNA is bound to RIG-I only via the CTD, and the helicase-mode, where the dsRNA is bound by the CTD and helicase domains (Figure 4). The helicase-mode complex is required for breaking the CARD2:Hel2i interactions to activate RIG-I signaling pathway via the exposed CARDs. While there are many high-resolution structures of the helicase-mode conformation, the structure of a CTD-mode conformation of RIG-I has not been determined.

## RIG-I proofreads RNA via an ATPase-driven translocation mechanism

**Figure 5.**
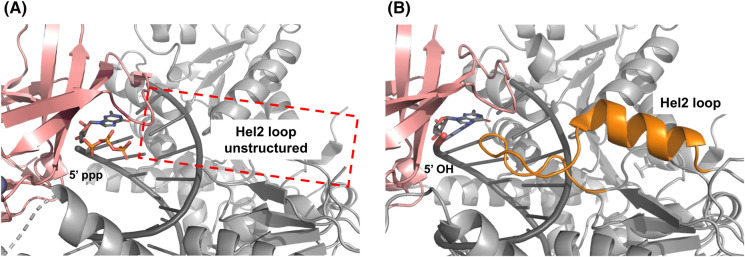
An unstructured Hel2 loop is a signature of RIG-I translocation throttling. (**A**) The Hel2 translocation loop is unresolved in the structure of RIG-I bound to 5′ppp dsRNA (PDB: 5F9H), signifying the long-lived translocation throttling state. (**B**) Resolution of the Hel2 translocation loop (orange) in the structure of RIG-I bound to 5′-OH dsRNA (PDB: 5F9F) indicates the propensity for this complex to rapidly dissociate via translocation.

The RNA binding specificity of the CTD and the CHL gating mechanism in RIG-I significantly reduce the likelihood of RIG-I binding to non-specific RNAs. If RIG-I engages non-specifically with RNAs, its ATPase-driven translocation activity adds an extra layer of RNA proofreading to dissociate RIG-I from non-specific RNAs [35,37] (Figure 4). The Hel2 loop in Helicase motif IVa (664–685) plays a key role in RIG-I translocation [31]. This region remains unresolved in RIG-I structure bound at the 5′ppp RNA end [59,62] (Figure 5A) but is structured when RIG-I is bound to RNA internally or to non-ligand RNA ends, like 5′-OH [37,62] (Figure 5B). Thus, as RIG-I breaks its interactions with the 5′ end, the mobile state is characterized by the formation of the Hel2 loop that interacts with the major groove of the RNA to promote translocation. Mutations in the Hel2 loop (T667E and T671E) or deleting the α-helix portion of the Hel2 loop region, reduces translocation activity and signaling ability [37].

The ATPase-driven translocation activity differentially affects the binding of RIG-I to 5′ppp and non-specific RNAs. When RIG-I binds to a 5′ppp blunt-ended dsRNA, the interactions between the CTD and the 5′ppp are strong and they anchor RIG-I to the RNA end, slowing down its translocation away from the 5′ppp end. As a result, RIG-I remains more stably bound to the 5′ppp RNA [37]. This ‘translocation-throttling' action by the CTD increases the lifetime of CARDs-exposed RIG-I on the 5′ppp RNA under physiological ATPase conditions. In contrast, the CTD has a low affinity for RNAs without a 5′ppp or blunt end. As a result, it does not impede RIG-I's movement along these types of RNAs, and ATP hydrolysis quickly dissociates RIG-I from non-specific RNAs (Figure 4). Thus, the ATPase-driven translocation activity contributes to RNA proofreading and decreases the lifetime of activated RIG-I on non-specific RNAs.

The ATPase-driven translocation serves not only to dissociate RIG-I from non-specific RNA ligands but also to regulate the duration of its activated state on viral RNAs. Despite the threat of viral infection, sustained signaling can harm the host. Therefore, it is crucial to dynamically associate and dissociate from RNAs and control the lifetime of RIG-I on viral RNA, a process driven by its ATPase activity. In vitro studies show that non-hydrolyzable ATP analogs enhance RNA binding and prolong the time RIG-I remains bound to both 5′ppp and non-specific RNAs [37]. RIG-I variants with point mutations in the ATPase active that render RIG-I unable to hydrolyze ATP are linked to chronic inflammatory conditions like Singleton Merten syndrome (SMS) [19]. This sustained immune activation in the SMS RIG-I variants is caused by their binding to non-specific endogenous RNAs. Biochemical studies reveal that SMS mutants form stable complexes on non-specific RNAs in the presence of ATP [37,54,73].

The ATPase and translocation functions of RIG-I also promote RIG-I multimerization on long dsRNA [37,61]. When the end-bound RIG-I molecule moves internally, the exposed blunt end and 5′ppp becomes available for another RIG-I molecule to bind (Figure 4). This multimerization of RIG-I on dsRNA may facilitate the subsequent tetramerization of the CARDs domains and polyubiquitination by the E3 ligase RIPLET, as studies suggest that RIG-I dimers have a higher affinity for RIPLET [74]. The covalent and non-covalent interactions of the K63-linked polyubiquitin chains of RIG-I CARDs are essential for MAVS activation leading to downstream signaling events [10,63,75–79].

## Host evasion strategies: RIG-I's awareness for m7G Cap-1, 5′-monophosphate, and non-blunt end RNAs

RIG-I is present in the cytoplasm of most cells where there is a vast pool of RNAs that must be avoided [20–22]. The high specificity of RIG-I CTD for the 5′ppp blunt-ended dsRNA and its ability to discriminate against other RNA ends plays a crucial role in RNA discrimination. Additionally, host cells hide the RNA ends from RIG-I recognition via post-transcriptional RNA modifications, including RNA capping and ribose methylation, generation of RNA overhangs, and 5′-monophosphate RNA.

### 7-Methyl guanosine RNA capping

**Figure 6.**
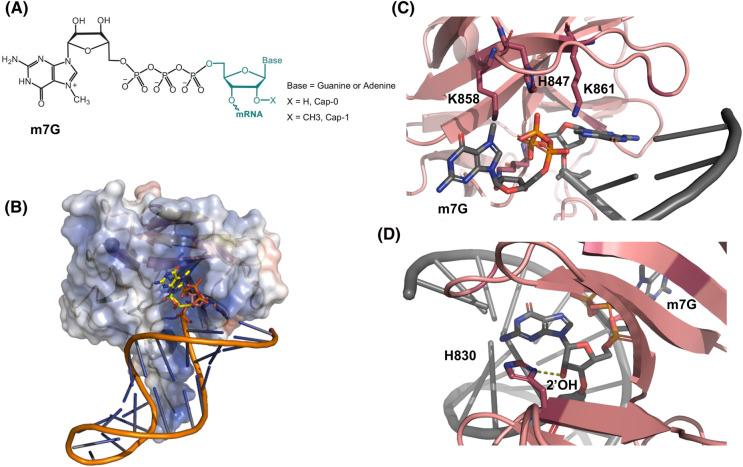
Host mRNA capping and ribose methylation (Cap-1) are essential for evading recognition by RIG-I. RIG-I must avoid engagement with host mRNAs present in the cytoplasm. (**A**) The distinguishing features of mRNA capping include an m7G moiety (Cap-0) and a 2′-*O*-methylation of the end-nucleotide ribose (Cap-1). (**B**) RIG-I crystal structure (PDB: 5F98) bound to 5′ cap-0 dsRNA shows the CTD surface electrostatics accommodating the bulky m7Gppp moiety. (**C**) 5′ triphosphate and base-stacking interactions are maintained by the CTD capping loop with the terminal base pair of the Cap-0 dsRNA. (**D**) H830 interacts with the 2′OH of the ribose of the 5′-end nucleotide in dsRNA. The H830 is expected to clash with the 2′O-Me of the ribose, allowing RIG-I to discriminate against Cap-1 mRNA. Mutation to H830A abolishes this checkpoint entirely.

When cellular RNAs are transcribed by Pol I, Pol II, Pol III, and mitochondrial POLRMT, they possess a common feature — a 5′ppp end that has the potential to be recognized by RIG-I. However, most RNAs with these features are compartmentalized and protected from RLR access and undergo co-transcriptional or post-transcriptional modifications before entering the cytoplasm. Specifically, Pol II mRNAs undergo transcriptional modification at their 5′ppp end through capping and methylation reactions [80]. In the case of Pol II mRNAs, the 5′ppp end is capped with guanosine and then methylated at the N-7 position, resulting in Cap-0 RNA with a m7G cap that is necessary for recognition by the translation initiation factor eIF4E in the ribosome machinery [80]. Interestingly, in vertebrates (but not yeast), the ribose of the first and second nucleotides from the capped end have a 2′-*O*-methylation (Cap-1 and Cap-2, respectively) (Figure 6A). Mitochondrial RNAs do not contain an m7G cap but can form double-stranded structures and can end up in the cytoplasm and activate the RLRs [81].

Biochemical and structural studies have shown that an m7G cap itself in dsRNA does not hinder RIG-I detection. Cap-0 RNA and 5′ppp RNA have nearly identical binding affinities to RIG-I (Table 1), activating its ATPase and cellular signaling responses to similar extents [59]. HDX-MS experiments also show that Cap-0 RNA binding to RIG-I induces the same conformational change that leads to CARD exposure [54]. Structural studies of RIG-I bound to Cap-0 RNA reveal that the CTD domain can accommodate the bulky m7G cap moiety while maintaining interactions with the triphosphate [59] (Figure 6B,C).

**Table 1.** RIG-I RNA end-binding affinities

RIG-I RNA end-binding affinities							
		Ref. [68]		Ref. [59]		Ref. [93]	
10 bp hairpin RNA	*K*_D_^1^ (nM)	23 bp dsRNA	*K*_D_ (nM)	10 bp hairpin RNA	*K*_D_ (nM)	12 bp dsRNA	*K*_D_ (nM)
5′ ppp	4.6	5′ ppp	0.8	5′ ppp	1.8	5′ ppp	1.42
5′ OH	6.1	5′ OH	7.8	5′ OH	38.5	5′ pp	1.86
5′ ppp 3′ ovg	13.7	5′ ppp 3′ ovg	9	5′ Cap-0	1.7	5′ OH	13.1
5′ ppp 5′ ovg	18.5	5′ ppp 5′ ovg	87	5′ ppp 2′-O-Me	40.0	5′ p	49.3
5′ OH 3′ ovg	187	5′ OH 3′ ovg	336	5′ Cap-1	425		
5′ OH 5′ ovg	307	5′ OH 5′ ovg	420				
5′ p 3′ ovg	203						
Stem dsRNA	1250						

RNA *K*_D_ values from select publications are summarized.

1 Unpublished data.

In contrast, Cap-1 modifications involving 2′-*O*-ribose methylation are not tolerated and disrupt RIG-I binding and signaling [59,82]. This disruption is linked to a mechanism involving the conserved H830 residue in the CTD of RIG-I, which interacts with the 2′-OH of the 5′-end nucleotide ribose (Figure 6D). The structures suggest that the 2′-*O*-Me would clash with H830, accounting for the significantly weakened RNA-binding affinity. The m7G capping with 2′-*O*-methylation synergistically weakens the RNA affinity of the isolated CTD and full-length RIG-I by 100- to 200-fold (Table 1). RIG-I with an H830A mutation shows an increased affinity for Cap-1 RNA and aberrant activation by cellular RNAs [59,82]. Thus, the conserved H830 residue in RIG-I's CTD is a sensor of 2′-*O*-Me modifications in RNA and a crucial discriminator between self and non-self-RNAs. Many viruses, such as SARS, Dengue, Ebola, and Marburg, encode both capping and 2′-*O*-methyltransferase activities to evade RIG-I. Therefore, targeting their 2′-*O*-methyltransferase activity may emerge as an effective strategy for future antiviral therapies, as has been demonstrated with both flavivirus and coronavirus methyltransferases [83–85].

Similarly to RIG-I's accommodation of the bulky m7G cap, a recent study has identified a new class of 5′ metabolite-capped dsRNA RIG-I ligands, binding with affinities similar to 5′ppp RNA and stimulating the RIG-I signaling pathway [86]. NAD^+^, FAD, and dpCoA metabolites can be incorporated as initiating nucleotides during DNA transcription instead of ATP [87,88]. The mitochondrial RNA polymerase, in particular, can use NAD^+^ and NADH to initiate transcription [87]. Metabolite-capping was also found in Dengue viral genomes [89]. The metabolite-capped RNAs contain a diphosphate bridge instead of a triphosphate, as in m7G capping. However, it appears that RIG-I can accommodate the bulky size of the metabolites while maintaining interactions with the diphosphate moiety.

### Non-blunt-ended RNA

The blunt end in dsRNA is recognized by stacking interactions of F853 and F856 in the CTD of RIG-I. The identity of the terminal blunt end base pair does not matter; however, terminal mismatches are not tolerated [90], and the affinity is reduced drastically when the dsRNA contains an overhang at the 5′- or 3′-end [53,68,86,90]. Biochemical studies have determined that the 3′-overhang with 5′ppp has a higher affinity for RIG-I than the 5′-overhang with ppp (Table 1), and the former can also weakly activate RIG-I signaling [68]. Modeling studies suggest that the 3′-overhang can be accommodated in the RNA-binding channel of the CTD while the 5′-overhang clashes [31]. Without 5′ppp, both types of overhang RNAs bind weakly and do not activate RIG-I signaling. Arenaviruses have a 5′-triphosphate group on their genomes, but the transcription mechanism creates an atypical 5′-nucleotide overhang, which provides an advantage to the virus by deterring RIG-I recognition [91].

### 5′-Monophosphate RNA end

5′-p RNA ends in the cell arise from RNA cleavage reaction during various biological processes, including post-transcriptional processing of rRNAs from the Pol I reactions, tRNAs from the Pol III reactions, and processing of mitochondrial RNAs. Additionally, the dsRNAs of the cellular silencing mechanisms, such as microRNA (miRNA) and small interfering RNA (siRNA), abundantly present in the cell, contain a 3′-overhang and 5′-p. Interestingly, RIG-I has a mechanism to discriminate against the 5′-p RNA end. Studies show that the binding affinity of RIG-I for 5′-p dsRNA is ∼5-fold less than 5′-OH RNA and 30-fold less than 5′-ppp dsRNA (Table 1) [90,92,93]. This is particularly relevant as many viruses, including Hantaviruses, process their RNA ends to remove the 5′ppp end nucleotide, leaving a 5′-p, which would weaken RIG-I recognition [94].

A recent study identified the I875 residue in the CTD domain of RIG-I as a discriminator of 5′-p RNA end [92]. As discussed earlier, RIG-I utilizes its CTD domain to survey RNAs, and this study shows that the isolated CTD has an extremely low affinity for 5′-p RNA (1.5 µM) compared to the 5′ppp RNA (0.05 nM) or 5′-OH RNA (100 nM). This indicates that RIG-I discriminates against the 5′-p RNA end at the initial stages of RNA binding, forming a weak initial complex that presumably dissociates before the RNA is loaded into the helicase domain. However, on the off chance that RIG-I engages with the 5′-p RNA, the Hel2 loop remains structurally defined [62] (Figure 6C), indicating a lack of the throttling effect at the 5′-p RNA end. Thus, it is expected that the ATPase-driven translocation would quickly dissociate RIG-I from 5′-p RNA as it does for 5′-OH RNAs [37].

**Figure 7.**
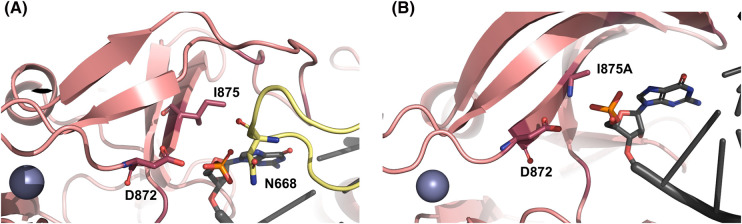
RIG-I discriminates against 5′ monophosphate dsRNA. There is an abundance of 5′ monophosphate dsRNA in the cell that RIG-I must actively discriminate against, including microRNA, tRNA, and leaky mitochondrial RNA transcripts. (**A**) The 5′-p dsRNA bound to the CTD of WT RIG-I shows potential interactions of 5′-p with a subset of lysines in the CTD capping loop (PDB: 7TNZ), yet RIG-I is not activated by 5′-p dsRNA. (**B**) The 5′-p dsRNA bound to the CTD of I875A RIG-I (PDB: 7BAI). No structural differences between panels **A** and **B** are apparent, although I875A mutation rescues RIG-I activation by 5′-p dsRNA.

Mutational studies indicate that introducing an I875A mutation in RIG-I restores 5′p dsRNA binding (160 nM) to a level similar to 5′-OH RNA and IFN response from 5′-p dsRNA. Interestingly, endogenous I875A RIG-I shows constitutive immune stimulation by cellular RNAs, underscoring the significance of residue I875 in the discrimination between self and non-self-RNA molecules. Curiously, structures of wild-type CTD and I875A CTD are very similar [65,92] (Figure 7A,B), and the cryo-EM structure of full-length RIG-I with 5′-p dsRNA shows no steric hindrance of I875 with the 5′-p moiety [62]. The structure shows the proximity of 5′-p to D872 and N668, which, when individually mutated to alanine increases IFN response from 5′-p RNA relative to wild-type RIG-I [93]. Thus, active discrimination against 5′-p may involve collective hindrance from I875, D872, and N668 amino acid side chains.

## Viral mechanisms to avoid detection by RIG-I

In response to the host's efforts to accurately detect and respond to viral RNA, viruses have evolved sophisticated evasion strategies to avoid detection by RIG-I and other host immune sensors. One such strategy involves masking RIG-I's activation ligand. The Ebola virus VP35 protein binds dsRNA in an end-capping mode [95], which can potentially interfere with RIG-I recognition, explaining its role in blocking the downstream interferon activation [96]. A recent study showed that the NS1B protein of influenza B virus competes with RIG-I for specific binding to 5′ppp dsRNA ends [97]. By sequestering the molecular signatures of RIG-I's RNA ligands, viral proteins may effectively inhibit RIG-I activation and dampen the host antiviral response.

Many viruses employ proteases to cleave key proteins in the RIG-I signaling pathway. HIV-1 protease directly targets RIG-I by mediating its sequestration to lysozymes for degradation [98]. There are many examples of viruses targeting MAVS as this protein is central to RIG-I signaling [99]. For example, the Hepatitis C Virus protein NS3-4A cleaves MAVS to dissociate its N-terminal region containing the CARD from the mitochondria to interrupt downstream signaling [100,101]. Several viral proteases also target IRF3, the transcription factor activating IFN [102]. A recent study has identified a new viral evasion system directly affecting RIG-I [103]. The SARS-CoV-2 protein Nsp5 cleaves RIG-I at the N-terminal CARDs, putatively removing 10 amino acids, preventing downstream IFN activation. Furthermore, Nsp5 promotes the ubiquitination of MAVS for proteasome-mediated degradation. These mechanistic examples highlight the ongoing evolutionary arms race between viruses and the host immune system, underscoring the importance of understanding viral evasion strategies for the development of effective antiviral therapies.

## RIG-I activation mechanism on long and short RNAs

Studies show that RIG-I preferentially binds to 5′ppp blunt-ended dsRNA and gets activated by a range of dsRNA lengths [66–69]. This poses an intriguing question regarding potential mechanistic differences in longer vs shorter dsRNA activation of RIG-I, and the implications in their downstream signaling capacities. Structurally and biochemically, there is no evidence suggesting a difference in the mechanism of activation for individual RIG-I molecules engaging with 5′ppp RNA end on short vs. longer ligands. RIG-I binds to the short 10 bp hairpin RNA as a monomer, whereas longer 5′ppp dsRNA (>19 bp) can accommodate multiple RIG-I molecules [37,38,53,67]. A 14-bp 5′ppp hairpin RNA induces a rapid IFN response [104], which suggests that RIG-I oligomerization on dsRNA is not essential for IFN response. A key conformational change in RIG-I resulting from 5′ppp RNA binding is disruption of the CARD2:Hel2i interface leading to CARDs exposure. This conformational change has been observed through HDX-MS, FRET, and NMR studies on RIG-I bound to 10- and 14-bp 5′ppp hairpin RNA and a 19-bp dsRNA [54,55,57,105]. The different modes of binding short and long dsRNAs may not affect the CARDs exposure on the RIG-I molecule bound to the 5′ppp RNA end but can influence the efficiency of the CARDs tetramerization step, required to nucleate MAVS filament formation.

**Figure 8.**
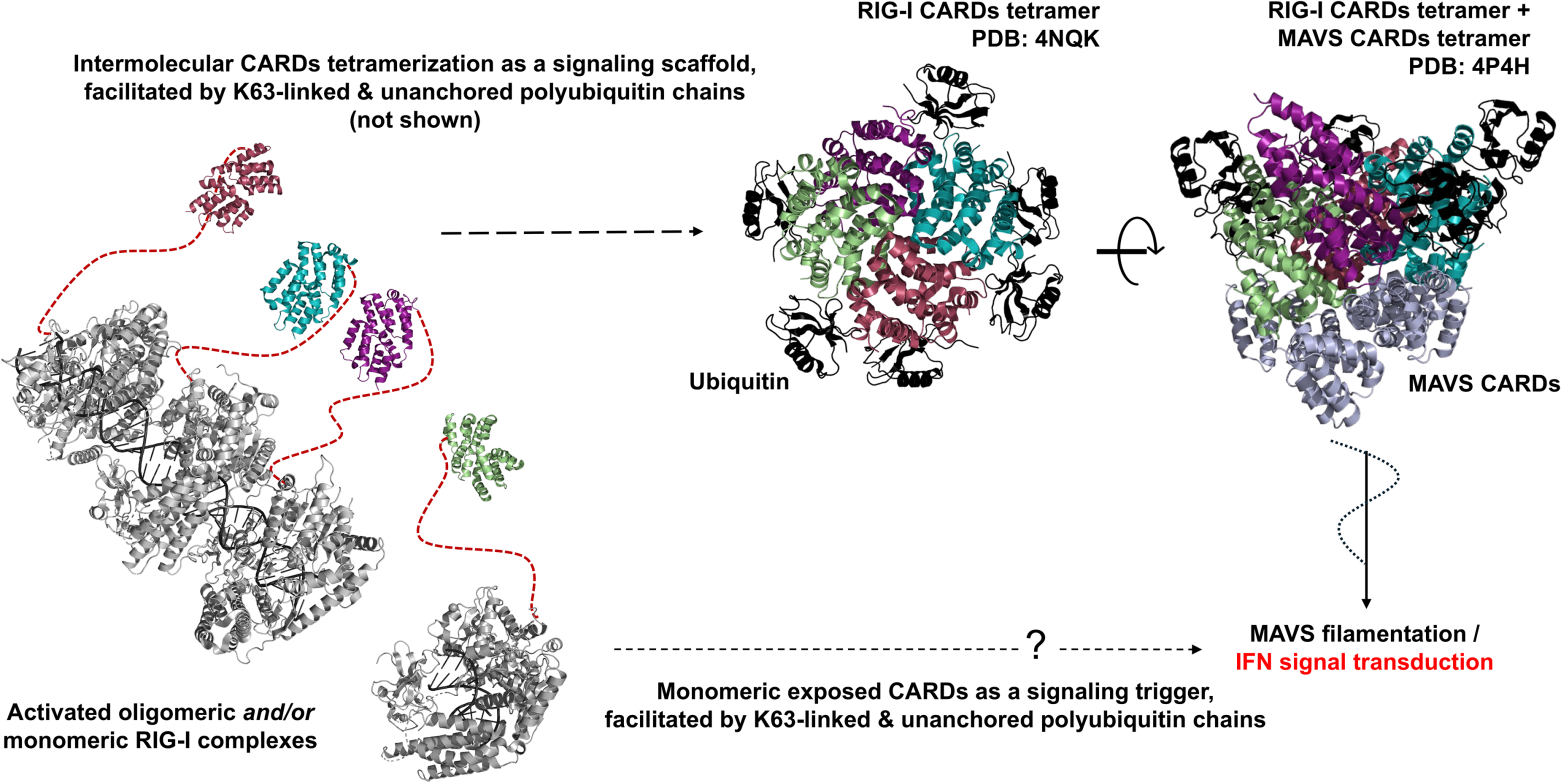
RIG-I signal transduction is mediated by CARDs tetramerization. The crystal structure of RIG-I CARDs reveals a tetrameric lock-washer formation, stabilized by K63-linked di-ubiquitin chains (PDB: 4NQK). Experimental results and an extended structure (PDB: 4P4H) of this formation suggest this scaffold is necessary to nucleate MAVS filamentation and activate downstream signaling events. RIG-I either clusters on long dsRNA (PDB: 7JL3) or intermolecularly across RNA ligands (PDB: 5F9H) to facilitate CARDs tetramer formation. Localized CARDs can interact with each other, and ubiquitination events stabilize these interactions.

There is strong evidence that exposed CARDs associate with polyubiquitin chains non-covalently or covalently to form a tetrameric structure that activates MAVS filament formation [10,49,50,75,77,79]. Crystal structures of RIG-I CARDs show a tetrameric lock-washer conformation (PDB: 4NQK) stabilized by non-covalent interactions with di-ubiquitin [49,50] (Figure 8). The lock-washer conformation of the RIG-I CARDs tetramers creates a nucleation site for MAVS CARDs to bind and initiate oligomerization (PDB: 4P4H). Thus, CARDs tetramers are an important checkpoint in the signaling pathway of RIG-I; however, direct evidence is lacking to show that these tetramers form in the context of full-length RIG-I. However, in support of the tetramerization model, specific mutations in the CARDs disrupting CARDs tetramerization ability also abrogate full-length RIG-I's signaling capacity [49].

There are two possible pathways for RIG-I CARDs to tetramerize on short and long dsRNAs. The CARDs may interact intermolecularly within the same RNA-bound complex, or across different RNA ligands (Figure 8). Following the assumption that internally bound RIG-I molecules maintain exposed CARDs, longer RNA ligands pose an obvious advantage in amplifying RIG-I's signal transduction by cooperative proximation of multiple activated molecules. In contrast, shorter RIG-I ligands may require a higher local concentration and polyubiquitination to proximate the same number of RIG-I CARDs for interaction across different RNA ligands. This logic favors the longer RNA as a more potent ligand for RIG-I at low RNA concentrations. A study showed that the negative impact of ubiquitin interaction mutations was partially alleviated by increasing the length of the 5′ppp dsRNA [49]. Thus, longer dsRNA ligands may have a looser requirement for K63-linked polyubiquitination for CARDs tetramerization than the short hairpin RNAs [78]. Recent evidence indicates that the E3 ligase RIPLET binds to a dimer of RIG-I on dsRNA with high affinity (6.6 nM), whereas interactions with RNA-bound RIG-I monomers or free RIG-I are over 100-fold weaker [74]. This difference in affinity may be due to RIPLET's self-association as a dimer, allowing it to readily bind to RIG-I dimers on longer dsRNA without needing to bring together two RIG-I monomers bound to short RNAs. Therefore, short and long dsRNAs may influence the efficiency of steps downstream from RNA binding to influence the IFN response.

*In vitro* studies suggest that RIPLET can also cluster RIG-I molecules bound to different RNAs and form large aggregates [78]. Whether large aggregates of RIG-I are necessary for activating MAVS is not clear. A cellular fluorescence microcopy study failed to detect aggregated RIG-I puncta near the mitochondria after activation with short hairpin signaling RNAs [104]. A super-resolution microscopy study suggested that signaling active MAVS filaments are ∼80 nm in size, containing ∼156 MAVS molecules [106], which could be sufficient for generating a platform for activating the downstream kinases and E3 ligases.

## Role of LGP2 in RLR regulation

An uncontrolled, sustained, or overly strong activation of the IFN response following a viral infection can be damaging. Thus, the antiviral signaling response is regulated at multiple steps [3] and the RLR member LGP2 has been shown to positively and negatively regulate the IFN responses of RIG-I and MDA5 [2,46,107–110]. At low concentrations, LGP2 activates MDA5 assisting in the early stages of viral recognition by enhancing the initial binding of MDA5 to the viral RNA and promoting filament assembly [107]. The LGP2 mixed filaments of MDA5 are shorter than filaments containing only MDA5 but generate equal or greater cellular signaling response [107]. LGP2 acts as a negative regulator of RIG-I and may compete with RIG-I for RNA binding [38]. However, LGP2 also inhibits RIG-I signaling independent of its RNA-binding activity [110]. LGP2, like RIG-I and MDA5, is an interferon-stimulated gene and when overproduced provides feedback inhibition to control the antiviral response. The exact mechanisms of feedback inhibition are not entirely clear. One study revealed that LGP2 interacts with the MAVS protein complex, blocking interactions with IKKε [47]. Other studies suggest that LGP2 associates with E3 ligases TRIM25 [109], TRAF [108], and the E2 enzyme, Ubc13/UBE2N [110] to inhibit the K63-linked polyubiquitination reactions. Despite these recent advancements in research, the molecular mechanisms behind LGP2's activities remain elusive. However, it is apparent that LGP2's intricate roles in RLR regulation, directly and through its feedback mechanisms, are critical in adjusting the level of antiviral and pro-inflammatory responses to viral infection.

## Summary

Each domain of RIG-I serves a specific function to ensure the accurate recognition of a viral dsRNA ligand. The CTD plays a crucial role in RNA selection throughout the multilayered RNA recognition and proofreading process, including initial RNA end selection and throttling RIG-I translocation to enhance its lifetime on viral RNAs. The open conformation of the helicase domains sequesters the CARDs in an autoinhibited state, with the disordered linker, CHL, stabilizing this state and ensuring that only RNAs favored by the CTD are loaded into the helicase. The double-edged ATPase activity of RIG-I proofreads for erroneous RNA binding and promotes the formation of signaling-competent complexes on pathogenic RNAs but also dissociates RIG-I and decreases its lifetime on viral RNA. Additionally, RIG-I has built-in sensors such as H830 and I875 that actively discriminate against Cap-1 and 5′-p RNAs found abundantly in the cytoplasm. Together, these mechanisms highlight the intricate and finely tuned processes by which RIG-I ensures accurate and effective recognition of viral dsRNA while minimizing off-target activation by endogenous RNAs.

## Future explorations

Current and future research is identifying critical steps to target for RIG-I activation and leading to the development of new assays for small molecule screening. This research opens up new possibilities in vaccine development, antiviral therapies, and treatments for autoimmune diseases and cancer immunotherapy. Designing molecules that can either modulate the ubiquitination steps or stabilize/destabilize CARD tetramers offers a way to directly control RIG-I activation. Treatment with RIG-I agonists generates a strong immune response that induces apoptosis and immunogenic cell death. This approach can work synergistically with existing cancer treatments [111–114], antivirals, and vaccine adjuvants antivirals [115–117]. Additionally, developing antagonists to suppress RIG-I activation could provide new therapeutics for chronic inflammation resulting from prolonged RIG-I activity [118,119] and treatments for diseases such as the Aicardi-Goutièrez Syndrome and SMS. Understanding and manipulating RIG-I signaling pathways offers a dual opportunity — enhancing immune responses for antitumor effects while managing inflammation for therapeutic benefits in diverse disease scenarios.

## Perspectives

- The intricate mechanisms by which RIG-I-like receptors (RLRs) discriminate viral RNA from endogenous RNA allow them to rapidly and accurately detect and respond to viral infections.
- Current research underscores the importance of RLRs, particularly RIG-I, in surveilling cytoplasmic RNA for viral infections. RIG-I employs a sophisticated array of mechanisms, including RNA end recognition, electrostatic gating, and ATPase-driven translocation, to ensure accurate detection of viral RNA while avoiding harmful immune responses to host RNA. RLRs modify host RNAs to evade recognition.
- Future investigations should delve deeper into RIG-I signal transduction's regulatory mechanisms, particularly the formation of CARD tetramers and subsequent MAVS filamentation. Understanding these processes at the molecular level holds promise for developing novel therapeutics, including vaccines and antivirals, synergistic approaches in cancer therapy, and strategies for modulating immune responses in autoimmune diseases.

## Abbreviations

CARDs: caspase activation and recruitment domains
CHL: CARDs-helicase linker
CTD: C-terminal end
DCs: dendritic cells
dsRNA: double-stranded RNA
LGP2: laboratory of genetics and physiology 2
MAVS: mitochondrial antiviral signaling protein
MDA5: melanoma differentiation-associated gene 5
NK: natural killer
RIG-I: retinoic acid-inducible gene I
RLRs: RIG-I-like receptors
SMS: Singleton Merten syndrome
